# Identifying CCR5 coreceptor populations permissive for HIV-1 entry and productive infection: implications for in vivo studies

**DOI:** 10.1186/s12967-022-03243-8

**Published:** 2022-01-24

**Authors:** Matthew Weichseldorfer, Yutaka Tagaya, Marvin Reitz, Anthony L. DeVico, Olga S. Latinovic

**Affiliations:** 1 Institute of Human Virology, School of Medicine, University of Maryland, 725 W. Lombard St., Baltimore, MD 21201 USA; 2 Department of Medicine, School of Medicine, University of Maryland, Baltimore, MD 21201 USA; 3 Department of Microbiology and Immunology, School of Medicine, University of Maryland, Baltimore, MD 21201 USA

**Keywords:** HIV, AIDS pathogenesis, CCR5 coreceptor populations, Conformational changes

## Abstract

**Background:**

The chemokine receptor CCR5 is the major coreceptor for HIV-1 cell entry. We previously observed that not all CCR5 mAbs reduce HIV-1 infection, suggesting that only some CCR5 populations are permissive for HIV-1 entry. This study aims to better understand the relevant conformational states of the cellular coreceptor, CCR5, involved in HIV entry. We hypothesized that CCR5 assumes multiple configurations during normal cycling on the plasma membrane, but only particular forms facilitate HIV-1 infection.

**Methods:**

To this end, we quantified different CCR5 populations using six CCR5 monoclonal antibodies (mAbs) with different epitope specificities and visualized them with super-resolution microscopy. We quantified each surface CCR5 population before and after HIV-1 infection.

**Results:**

Based on CCR5 conformational changes, down-modulation, and trafficking rates (internalization and recycling kinetics), we were able to distinguish among heterogeneous CCR5 populations and thus which populations might best be targeted to inhibit HIV-1 entry. We assume that a decreased surface presence of a particular CCR5 subpopulation following infection means that it has been internalized due to HIV-1 entry, and that it therefore represents a highly relevant target for future antiviral therapy strategies. Strikingly, this was most true for antibody CTC8, which targets the N-terminal region of CCR5 and blocks viral entry more efficiently than it blocks chemokine binding.

**Conclusions:**

Defining the virus-host interactions responsible for HIV-1 transmission, including specific coreceptor populations capable of establishing de novo infections, is essential for the development of an HIV-1 vaccine. This study hopefully will facilitate further development of inhibitors to block CCR5 usage by HIV-1, as well as inform future HIV-1 vaccine design.

**Supplementary Information:**

The online version contains supplementary material available at 10.1186/s12967-022-03243-8.

## Introduction

The history of HIV/AIDS antiretroviral therapies represents one of the most dramatic scientific struggles of the previous and current centuries. The outcomes of antiretroviral therapy, issues with drug induced adverse effects and the emergence of drug-resistant viruses could be improved with selective targeting of the CCR5 coreceptor subpopulations that are relevant for HIV-1 infection.

Primary HIV-1 infection is exclusively initiated by binding to CCR5, one of the families of G protein-coupled receptors, making it an attractive target for antiviral therapy. Regulation and identification of CCR5 populations on the cell surface are important parameters for determining the infection rates of HIV-1 [1–3]. The chemokine receptors consist of seven transmembrane helices, an extracellular N-terminus (NT), and three extracellular loops (ECLs). Epitopes located in the NT and second ECL (ECL2) of CCR5 are crucial for interactions with HIV-1, making them appealing options for blocking HIV-1 entry [4]. Some studies have shown that the NT domain is more critical for gp120 binding, while the extracellular loops (specifically ECL2) are more crucial for inducing conformational changes in the viral envelope that lead to membrane fusion and virus entry [4, 5]. Other studies have shown that HIV-1 gp120 may differ in the CCR5 regions to which they bind and have a more complicated binding pattern to different regions of CCR5 [6]. Recent studies have also demonstrated that gp120s from different HIV-1 strains could differentially explore the CCR5 transmembrane regions [7]. CCR5-tropic (R5) viral strains are by far the most prevalent and predominantly transmitted types [8, 9].

CCR5 has a crucial role during virus binding and entry, due to dynamic conformational changes that are modulated by its localization, trafficking, and G-protein association [10–12]. It was demonstrated that dimerization of CCR5 contributes to the conformational diversity of G-protein coupled receptors (GPCRs), which is a promising avenue of research for the prevention of HIV-1 entry [13]. Recognition of CCR5’s role has led to the development of effective antiviral drugs, including CCR5 antagonists and inverse agonists such as Maraviroc (MVC) [14–16] and fusion proteins that target the NT and other relevant sites in CCR5 [17, 18], as well as various anti-CCR5 antibodies [19–21].

Genetic polymorphisms of CCR5 have been correlated with HIV-1 resistance [22]. This is exemplified by individuals homozygous for the Δ32 deletion mutation of the *ccr5* gene. It encodes a non-functional protein that lacks regular surface expression and causes resistance to HIV-1 infection even after repeated exposures in homozygous individuals [23–28]. In addition, the most established phenotypic determinant of T/F viruses is CCR5 coreceptor dependency [29]. Interestingly, natural production of HIV-suppressive CCR5 ligands (e.g. RANTES) and genetic polymorphisms that modulate their expression are associated with resistance or control of HIV-1 infection [22]. Regarding interventions, the only known (albeit impractical) functional HIV-1 cure is bone marrow reconstitution with cells from a Δ32 CCR5 donor [30]. Moreover, CCR5 density levels (molecules/cell) on CD4+ T cells correlate with RNA viral loads [31] and progression to AIDS [32] in untreated HIV-1-infected individuals. The direct impact of CCR5 surface density on the antiviral activity of CCR5 antagonists was established by our group and others, showing that CCR5 levels inversely correlate with HIV-1 entry inhibition [1, 31, 33, 34], thereby suggesting that the potency of entry inhibitors is directly associated with, and dependent upon, CCR5 surface density [33, 35]. All of these and the successful curative impact seen from Δ32 mutation hematopoietic stem cell transplantation into *the Berlin patient* with AIDS and leukemia [36] have given additional impetus towards the use of CCR5 targeting drugs to combat HIV-1 entry and infection. Current anti-HIV therapies continue to be associated with long-term toxicity, drug-drug interactions, difficulties in long-term adherence, and elevated cost [37]. This study could contribute to new strategies towards designing CCR5 targeting drugs by reducing adverse effects and promoting drug adherence.

CCR5 also mediates cell migration and regulates cell activation during inflammation [38, 39], causing stabilization and then desensitization leading to its uncoupling from the G protein [12]. Chemokine ligands block virus binding sites and lead to surface CCR5 downregulation, which contributes to the anti-HIV activity of chemokines [8, 40, 41]. However, chemokine downregulation is specific to only certain subpopulations of CCR5 [42]. For example, CCR5 on T cells undergoes rapid chemokine-mediated internalization, while on monocyte-derived macrophages (MDMs), it does not, consistent with studies reporting that native chemokines are ineffective HIV-1 entry inhibitors on MDMs compared to T lymphocytes [38, 43], and demonstrating CCR5 subpopulation differences between cell types. In addition, CCR5 surface populations permissive for viral binding and entry may be altered after CCR5 trafficking or conformational changes, making the CCR5 trafficking pathway an attractive antiretroviral therapy drug target [40, 41, 44, 45]. Monoclonal antibodies (mAbs) specific for a single epitope can identify different coreceptor conformations and thus might be able to identify virus-permissive populations of CCR5. Some CCR5 antibodies remarkably suppress HIV-1 entry both in vitro and in vivo [19–21, 46], whereas other CCR5 antibodies do not.

Here we examined the function and dynamics of CCR5 conformational subpopulations and linked them with HIV-1 entry and productive infection in different cell types, including physiologically relevant donor cells. In addition, we identified the CCR5 conformational subpopulations that allow cells to become permissive to productive HIV-1 infection. Hopefully this will lead to the identification of better HIV-1 entry inhibitors of prophylactic or therapeutic efficacy in the future.

## Materials and methods

### Cell culture

JC10 cells (HeLa derivative, low CCR5 expression; ~ 2,000 mols/cell) and JC53 cells (HeLa derivative, high CCR5 expression; ~ 130,000 mols/cell) were generated and maintained as described in Platt et al. [47], and PBMCs were obtained from the MicroQuant Leukopaks at IHV UMSOM. JC10 cells, JC53 cells, and PBMCs were cultured in RPMI-1640 with 10% FBSΔ, l-glutamine, and antibiotics. PBMCs were stimulated with 5 µg/mL PHA and 50 UI/mL IL-2 for 3 days prior to an experiment. U87.CD4 cells were cultured in Opti-MEM with 15% FBSΔ and without phenol red.

### Immunostaining

Cells were transfected with plasmid expressing CCR5-GFP (or CCR5-HA), with GFP or HA fused to the C-terminal (CT) region of CCR5, using a Genepulsor (Bio-Rad) at 270 V (JC10, JC53) or 170 V (U87.CD4)/960µF. Post-transfection, cells were cultured for an additional day and tagged with mAbs against extracellular CCR5 regions. JC10 and JC53 cells treated with FLSC IgG1 received 5 µg/mL for 20 min at RT prior to surface primary antibody labeling. Cells were then permeabilized with eBioscience Permeabilization Solution (Cat. 00-8333-56) for 5 min at RT (JC53) or 0.01% Triton X-100 in PBS for 20 min at 4 °C (U87.CD4, PBMCs) prior to intracellular staining. Each group was stained with one of 1:10 T 21/8 (Invitrogen, Cat. 14-1957-82), CTC5 (R&D Systems, Cat. MAB1802), CTC8 (R&D Systems, Cat. MAB1801), 2D7 (BD Biosciences, Cat. 555991), 45523 (R&D Systems, Cat. MAB181), or 45531 (R&D Systems, Cat. MAB182) CCR5 primary antibodies in PBS with 1% FBSΔ for 20 min at RT, followed by 1:500 anti-mouse AlexaFluor 647 (abcam, Cat. ab150107) under the same conditions. PBMCs were additionally labeled with 1:150 anti-CCR5 C-terminus (abcam, Cat. ab63123) in PBS with 1% FBSΔ for 20 min at RT, followed by 1:500 anti-rabbit DyLight 488 (Invitrogen, Cat. 35552) under the same conditions after permeabilization. Samples were fixed with 3:4 IC Fixation Buffer (Invitrogen, Cat. 00-8222-49) in PBS for 30 min, and then nuclei were stained with 50 µg/mL DAPI for 1 min at RT. Cells were imaged in HBSS using a Zeiss LSM 800 confocal system and Airyscan super-resolution (Carl Zeiss Microscopy, Germany). Cells were analyzed for CCR5 CT labeling (green) and CCR5 extracellular region mAb labeling (red), including the NT and ECL2 using ZEN 2.3 Blue Software. The CT label allows us to track and quantify intracellular and surface expression of CCR5, while also allowing coincident fluorescence imaging of the molecule by mAbs [12]. Such dual staining in 3D provides much greater precision than conventional confocal methods. The dual staining concept is based on previous publications that employed numerous fluorescently tagged mAbs against CCR5 extracellular domains and a CCR5-GFP CT to determine how conformation, localization, internalization, trafficking, and G-protein association are related to permissivity for viral entry [12]. Statistical analysis for surface vs total data sets was by Student’s t-test.

### Internalization of coreceptor

U87.CD4.CCR5-GFP cells were temperature shifted from 4 to 37 °C and incubated for 0, 15, 30, 45, 60, 75, or 90 min in culture media with 100 nM native RANTES or volume-equivalent control before fixation as described above [48]. After fixation, cells were washed, stained, and imaged as described above. Colocalization coefficients (see “Confocal image acquisition and analysis” section) were calculated as a percentage of the 0-min time point.

### Recycling of coreceptor

U87.CD4.CCR5-GFP cells were temperature shifted from 4 to 37 °C and incubated for 90 min with 100 nM native RANTES [48]. U87.CD4.CCR5-GFP cells were then re-suspended in culture media with 0.45 M sucrose, 1 µM cytochalasin D, or volume-equivalent control for 0 (directly after RANTES removal), 30, 60, or 120 min at 37 °C [12, 48]. Cells were fixed, washed, and stained as described above. Colocalization coefficients (see “Confocal image acquisition and analysis” section) were calculated as a percentage of the volume-equivalent control before graphing.

### Infectivity assay

Cryopreserved PBMCs were thawed and cultured/activated as described above. Cells were washed and incubated with HIV_BaL_ (MOI = 0.00075) and 50 UI/mL IL-2 for 3 h at 37 °C, 5% CO_2_, with control cells not challenged with HIV-1 [49]. Cells were washed and left in culture with 50 UI/mL IL-2 for 1 week with a half media change after 3 days. Cells were collected and stained as described above. Infected cells were examined for p24 expression via ELISA to verify the productive infection by HIV-1 (data not shown). Cell culture supernatant was collected and frozen at − 20 °C, and later thawed and added to a 96-well flat-bottom plate at a 1:200 dilution in 1× lysis buffer and culture medium. HIV-1 p24 was quantified using the PerkinElmer Alliance HIV-1 p24 Antigen ELISA Kit (Cat. No. NEK050001KT). Surface expression was measured as the colocalization coefficient and compared before and after infection to determine which conformations were primarily internalized by HIV-1 binding (Donor #7003). This assay was repeated using another donor (Donor #0872) with similar results (data not shown). Statistical analyses were evaluated by comparing relative surface expression (surface coefficient/mean total coefficient) using Student’s t-test to measure significance between infected and non-infected data sets.

### Confocal image acquisition and analysis

Three Airyscan (~ 120 nm resolution in x–y direction) laser lines at 405 nm (DAPI, violet), 488 nm (GFP, green) and 647 nm (mAb, far red) were used to excite fluorescence signals in these imaging experiments, which was separated by the quad DAPI/FITC beam splitter and acquired by the EM-CCD camera [50] via the Carl Zeiss LSM 800 confocal system (Carl Zeiss, Germany). Individual fluorescence signals were further acquired using a Gasp detector (Carl Zeiss LSM 800, Germany). A Plan-Apochromat 63×/1.4 oil DIC objective (Carl Zeiss LSM 800, Germany) was used to visualize three-colored cell samples. All parameters were consistent in each experiment, including laser excitation power and detector, and offset gain. ZEN Blue 2.3 software (Carl Zeiss Microscopy) was used to generate and analyze original images. Non-transfected cells were stained and imaged with the same conditions as described above (data not shown) and used as a negative control to calculate the background. All the images were acquired using the same instrumental settings. Signal to noise ratio was assured by measuring the total intensity of each image and averaging data for each image set. Oversaturated signal was avoided by using the software’s algorithm. Images were analyzed to determine the percentage of CCR5 conformations. GFP labels (green pixels) were used to calculate total (all surface and intracellular) CCR5 and overlapping staining (yellow pixels) was used to calculate primary antibody staining. CCR5 colocalization coefficients were calculated using the primary staining (yellow) divided by total CCR5 (green and yellow), for each monoclonal antibody detecting a different conformation of CCR5 (red signal). Colocalization coefficients calculate the percentage of total CCR5 receptors labeled by the mAb. Surface colocalization measures only mAb-bound CCR5 at the cell surface, while total colocalization measures mAb-bound CCR5 both at the surface and inside the cell. The difference between surface and total colocalization coefficients is the amount of intracellular only mAb-bound CCR5. Color thresholds and background were determined using single color controls processed through ZEN Blue 2.3 software. Values for data analysis were collected by compiling data from at least 10 randomly picked sample fields. GFP and mAb signals were collected as voxel fluorescence intensities.

## Results

### Visualization of distinct CCR5 epitopes by double staining

**Fig. 1:**
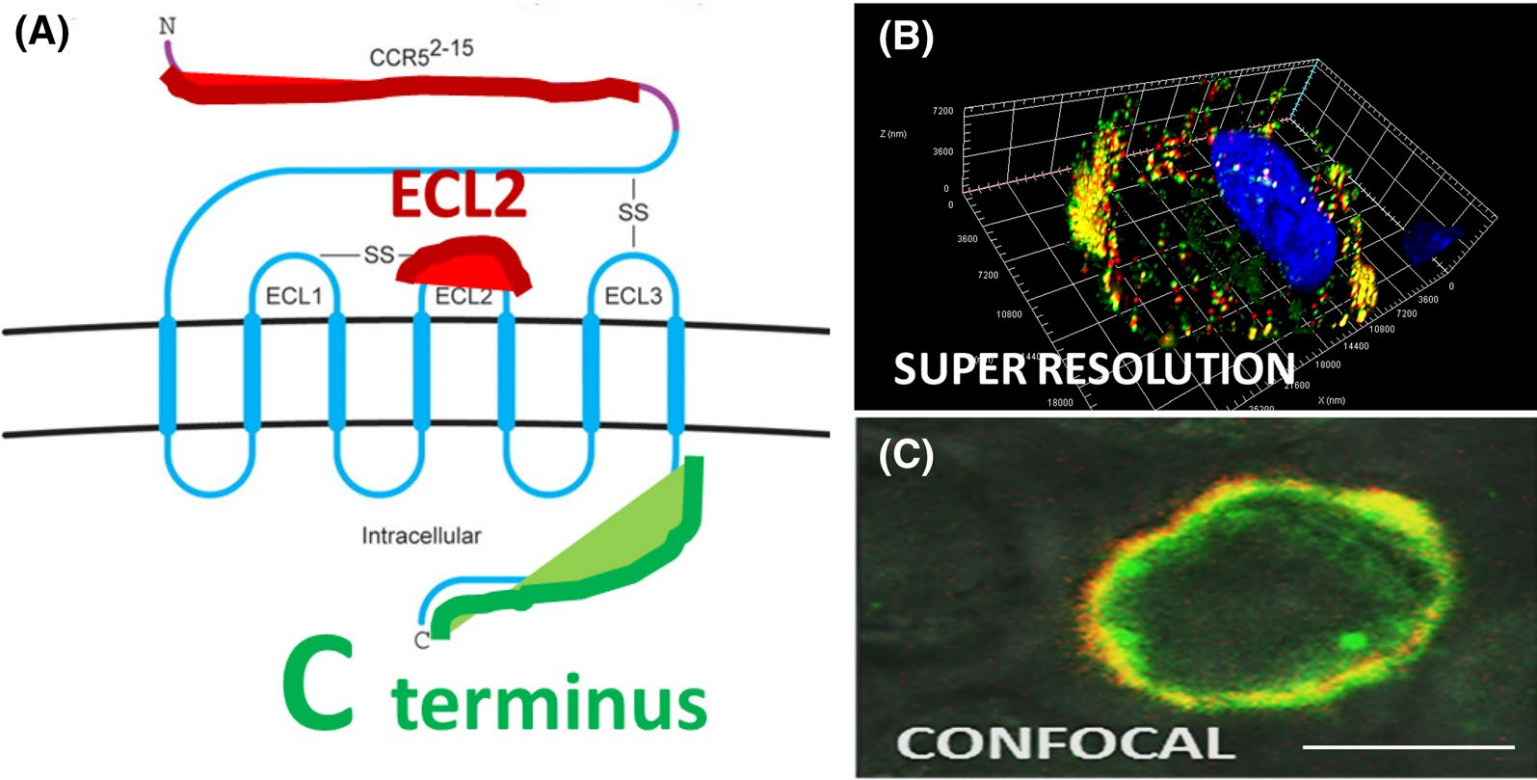
Comparison of dual staining CCR5 imaging methods. The C-terminus is GFP tagged (green); the extracellular epitopes are stained with domain specific mAbs (red) (**A**). A U87.CD4.CCR5-GFP cell expressing CCR5 C-terminus-fused GFP, stained with Alexa 647-conjugated anti-ECL1/ECL2 45523 mAb (**B**) [50] and a confocal image under the same conditions (**C**). Bar size—10 µm

The abundant presence of CCR5 on the cell surface is essential for HIV-1 binding, entry, and infection. We investigated the quantities and distributions of the different subpopulations of CCR5 in selected cell lines. Figure 1 shows the dual staining methodology that allowed us to visualize CCR5 events within the cellular spaces of cell lines and donor cells using ZEN Blue 2.3 software with comparative images between Airyscan-based super resolution and standard confocal microscopy. Figure 1A schematically shows the double labeling strategy used in our imaging experiments. The figure depicts the C-terminus-GFP fusion protein (green) and NT/ECL2 tagged with an Alexa 647-conjugated mAb (red). This Airyscan-based super-resolution technique (Fig. 1B) enabled greater resolution of CCR5 localizations and staining puncta versus standard confocal microscopy (Fig. 1C), which failed to provide more comprehensive results for this type of study [50]. This level of resolution permits quantitation of the total amount of CCR5 bound by mAbs on a cell as well as internalized events. Further, mAb-bound CCR5 on the cell surface versus intracellular compartments can be effectively distinguished by imaging samples of cells with and without membrane permeabilization.

To establish an imaging procedure for surface staining of CCR5, we first wanted to be able to visualize different levels of surface CCR5 conformations, as identified with different monoclonal antibodies, namely CTC8, labeling the CCR5 NT, and 2D7, labeling the CCR5 ECL2, in red color. The JC10 cell line expresses ~ 2,000 molecules per cell (Additional file 1: Fig. S1A) whereas the JC53 cell line expresses ~ 130,000 CCR5 molecules per cell (Additional file 1: Fig. S1B). There is a clear difference in the intensity levels of the surface CCR5 signal between the two cell lines in all sets of samples, as expected due to the difference in their natural surface CCR5 expression levels. Both cell lines showed a decrease in mAb binding to CCR5 when cells were pretreated with full length single chain IgG1 (FLSC IgG1), a chimeric protein containing HIV-1_BaL_ gp120 and the D1 and D2 domains of human CD4. FLSC IgG1 competitively binds the coreceptor at two different regions, the NT and ECL2 [17, 18]. As expected, the visualized CCR5 signal was correspondingly lower in the JC10 cell line due to lower CCR5 expression (Additional file 1: Fig. S1).

**Fig. 2:**
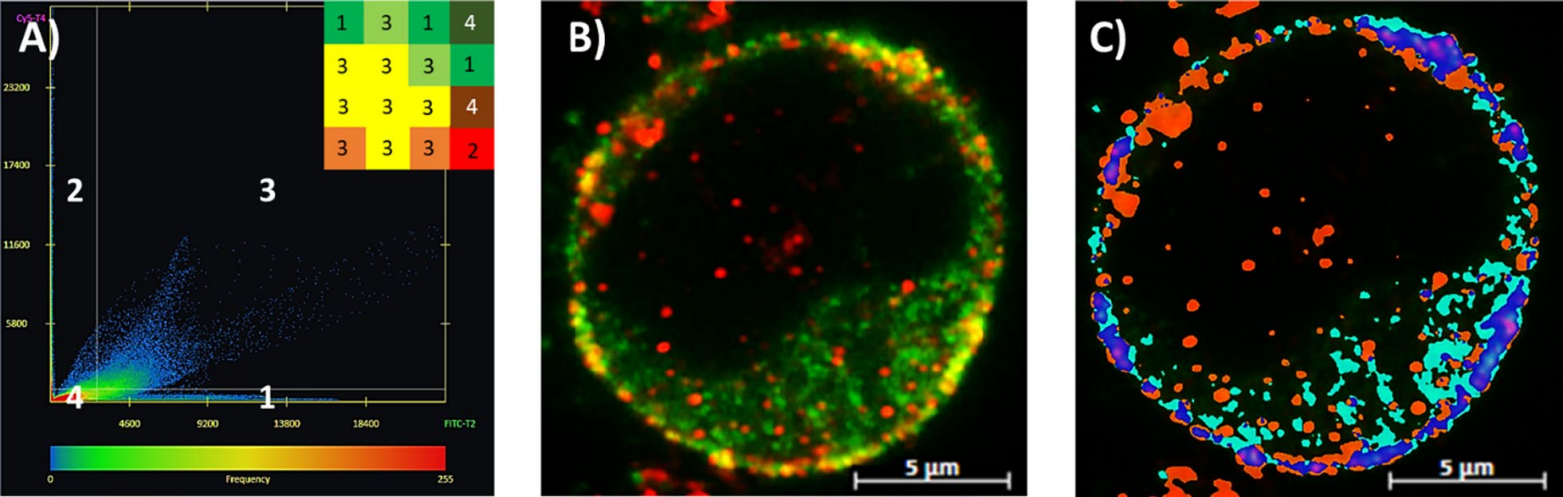
Colocalization analysis of CCR5 epitopes. A simplified example of how colocalization is quantified. When the NT or ECL2 CCR5 epitope antibody (red) labels a CCR5 C-terminus-GFP fusion protein (green), the overlapping fluorophores create a yellow signal. Unlabeled ectopically expressed CCR5-GFP fusion protein will show as green only and labeled endogenously expressed CCR5 will show as red only. The color identity and density spread are displayed in the graph along with the intensity thresholds set (white lines) using single color controls. The inset is a cartoon of which category a pixel within the image is assigned based on the thresholds, with 1 being green only, 2 being red only, 3 being yellow (overlap), and 4 being background (**A**). A cell (**B**) is analyzed using the ZEN Blue 2.3 software, and each of the colors are identified by the computer based on the parameters set. The extracellular CCR5 epitope in this example (NT; CTC8) is highlighted in orange, CCR5-GFP in cyan, and NT + C-terminus-GFP overlap in dark blue by the analysis software (**C**). The pixels in these identified regions are counted by the program, and the colocalization coefficient is calculated (dark blue/dark blue + cyan) to determine what percentage of CCR5-GFP was bound by the antibody. Bar size—5 µm

Figure 2 demonstrates how the ZEN analysis was used to quantify the colocalization of CCR5 events, allowing us to measure different CCR5 conformational subpopulations using our dual staining method. Figure 2A shows the pixel color spread and density along with the thresholds with which the program assigns color identity, determined using single color controls. The section labeled 1 is green color only (GFP), section 2 is red color only (Alexa 647-conjugated mAbs), section 3 is overlapping signal (high green and red, appears as yellow color), and section 4 denotes background (low green and red signals). Figure 2B is the Airyscan-based image of visualized dual CCR5 staining where the green signal shows the labeled CCR5 CT, while the red signal is extracellular and indicates NT or ECL2 labeling. The yellow signal corresponds to overlapping signals. The intracellular region with low/no labeling is the nucleus (not shown to better visualize and focus on the CCR5 events). Figure 2C is a color-coded image using the parameters set in Fig. 2A by the ZEN Blue 2.3 software and shows how it quantifies pixel count to calculate the colocalization coefficient. Red signal only (Alexa 647-conjugated mAbs) pixels are labeled in orange, green signal only (GFP) pixels in cyan and overlapping yellow color (single CCR5 molecule) pixels in dark blue. Figure 2 shows our method of analysis in JC53 cells, that was also used for all other cell lines and donor cells in the study.

### Visualized surface versus total CCR5 distribution patterns in cell lines

**Fig. 3:**
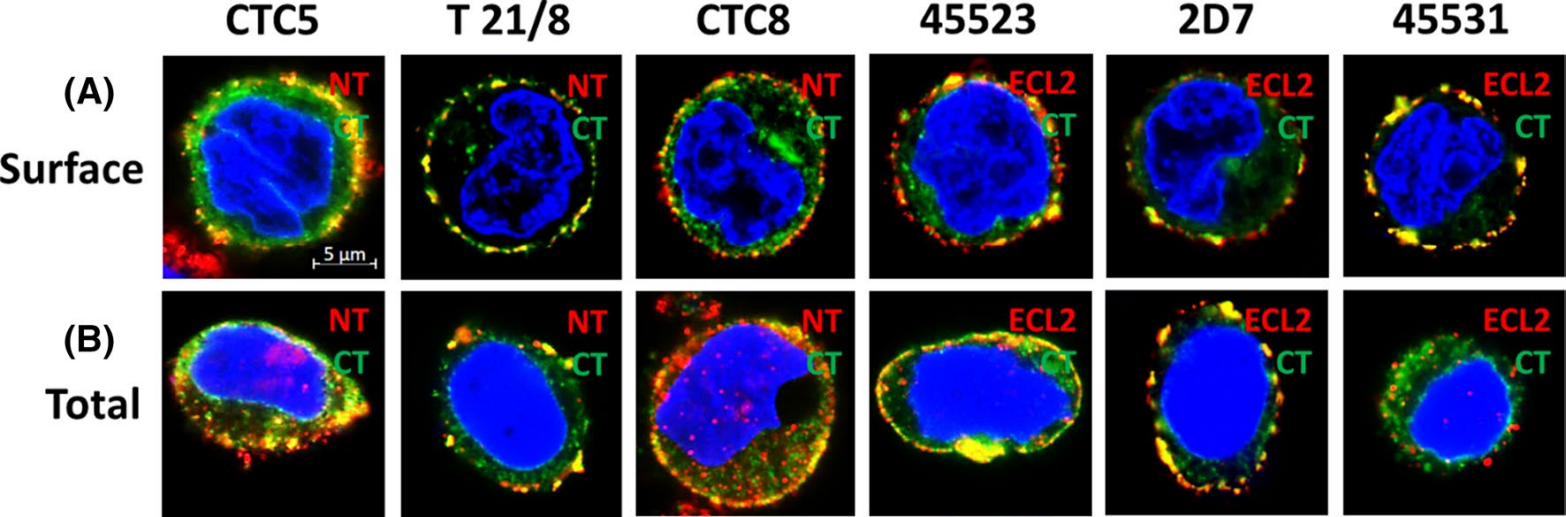
CCR5 conformation frequency visualized in JC53 cells. Quantified CCR5 subpopulation frequency in JC53 HeLa derivatives transfected with CCR5-GFP fused to the CT, showing total versus surface CCR5 events. Cells were fixed and labeled with an NT or ECL2 epitope-specific AlexaFluor 647-conjugated mAb (**A**). Cells labeled for total CCR5 events were fixed and permeabilized prior to staining (**B**). Cells were imaged using a Zeiss LSM 800 microscope and ZEN Blue 2.3 software. CCR5-GFP (green); mAb (red); co-localized signals (yellow). Bar size—5 µm

**Fig. 4:**
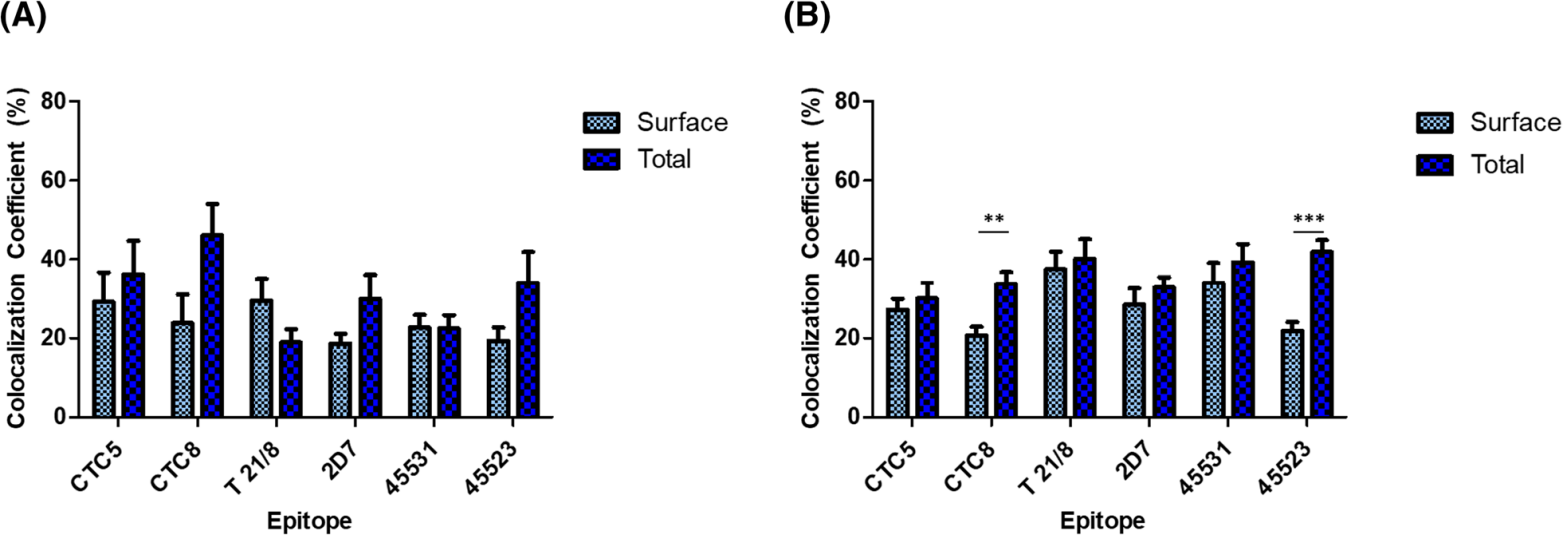
CCR5 conformation frequencies in different cell lines. JC53 (**A**) and U87.CD4 (**B**) cell lines were transfected with CCR5-GFP, grown in culture for 24 h post-transfection, and stained with one of six antibodies: CTC5 (NT), CTC8 (NT), T 21/8 (NT), 2D7 (ECL2), 45531 (ECL2), or 45523 (ECL1/ECL2). All primary antibodies were labeled with a secondary antibody conjugated with AlexaFluor 647. Cells were imaged and colocalization coefficients normalized to total GFP were calculated using ZEN Blue 2.3 software at ×1.3 zoom using 10 different cells. Data was graphed using GraphPad Prism 9. **p < 0.01; ***p < 0.001

We visualized the abundance, location, and distribution patterns of CCR5 conformations with different CCR5 epitopes exposed in the high CCR5-expressing JC53 cell line. To more precisely visualize and properly quantify CCR5 subpopulations with conformations permissive for viral binding and entry, we expressed CCR5-GFP fusion protein by plasmid electroporation into JC53 HeLa derivatives. Combined with the available CCR5 antibodies recognizing the NT and the ECL2 central epitopes, this allows two-color labeling to assess distances between epitopes. The C-terminus-GFP tag of CCR5 did not alter the intracellular and surface distribution of CCR5 (data not shown). The clear dynamics of CCR5 subpopulations and distinct pattern of inner (green signal; CT) versus outer (red signal; NT or ECL2) CCR5 membrane subpopulations were evident in the Airyscan-based imaging (Carl Zeiss, Germany). We visualized the surface versus total CCR5 subpopulations using six different mAbs. The images of panels containing mAbs against NT, ECL2, or against both [multiepitope (ME)] domains are presented in Fig. 3. Cultured cells were stained for surface CCR5 as described in “Materials and methods” section (Fig. 3A), then permeabilized prior to staining when measuring conformation frequency of internal CCR5 events to evaluate the presence or abundance of each conformation on the cell surface and within the cell relative to total CCR5 events (Fig. 3B). This yielded information on internal coreceptor conformations by comparing total and surface labeling percentages for each mAb of interest, that was later quantified. Visualizing total CCR5 subpopulations, we were able to determine whether most of each conformation is extracellular or intracellular. More detailed images of CCR5 conformational forms and divergent staining patterns are shown in Additional file 2: Fig. S2A with 2D7 (ECL2) visualized as a continuous pattern of puncta versus a distinctive clustered pattern, as seen with 45531 (ECL2, cholesterol-rich regions), while the quantified differences between surface densities are given in the graph of Additional file 2: Fig. S2B. Quantification of colocalization coefficients demonstrated that approximately 60% of 2D7 conformations were located on the surface whereas ~ 81% of 45531 conformations were located on the surface (Additional file 2: Fig. S2B), agreeing with the data in Fig. 4A. Punctuate staining patterns have been shown to be evident around dense cross-linked areas of actin filaments, which contribute to processes involved in virus entry, particularly endocytosis [51]. Airyscan based super resolution images in Fig. 3A, B show that the anti-ECL2 mAb 45531 has a more clustered staining pattern, most likely because it is binding to membrane regions that are rich in cholesterol [11, 12, 52], while the anti-NT mAb CTC5 yields more of a “capping” pattern, with CCR5 congregating on one side of the cell [38]. Interestingly, the patterns with the ME mAb 45523 or anti-NT mAbs are quite distinct from those of the anti-ECL2 epitope (2D7), which show a more uniform surface distribution pattern (Fig. 3A, B). These data agree with other findings by Flegler et al. [12].

### Quantified surface versus total CCR5 conformation frequency in cell lines

We next quantified the colocalization coefficient of total versus surface CCR5 events in the JC53 (Fig. 4A) and U87.CD4.CCR5-GFP (Fig. 4B) cell lines to compare CCR5 cellular distributions in a more physiologically relevant (U87.CD4.CCR5) cell line, and ultimately, primary cells. CCR5 was stained as described above and colocalization coefficients analyzed as described in Fig. 2. The first three sets in each Fig. 4 panel correspond to the mAbs against the NT: CTC5, T 21/8, and CTC8; the last three sets correspond to the mAbs against the ECL2: 45523, 2D7, and 45531, with 45523 being a ME mAb against both ECL1 and ECL2. Logically, the number of total events were higher than surface only using all mAbs, except for the T 21/8 mAb. The quantified data in Fig. 4A complemented the visual data of surface and total mAb staining of CCR5 events in Fig. 3. For example, the lack of visualized cell surface events seen with mAb 2D7 in JC53 cells (Fig. 3) was observed in the quantitative analysis (Fig. 4A) by its percentage labeling of surface CCR5 being the least among all mAbs. A similar pattern of staining with mAb 2D7 was also seen in U87.CD4 cells (Fig. 4B). Additionally, mAbs T 21/8 and 45531 show the lowest total staining both visually and quantitatively. Graphs of JC53 (Fig. 4A) and U87.CD4.CCR5 (Fig. 4B) surface vs total colocalization coefficients clearly show that some CCR5 conformations were more prominent inside the cell (ex. CTC8, 45523) while others were primarily localized to the cell surface (ex. CTC5, 45531). Additionally, cell type seemed to play a role in how conformations were distributed, with 2D7 more prominent on the surface in U87.CD4.CCR5 cells compared to JC53, where it was more heavily internalized. The following surface versus total percentages in the case of JC53 clones (anti-NT mAbs—CTC5, CTC8, T 21/8; anti-ECL2—2D7, 45531, 45523) were: 81%, 52%, 100%, 62%, 100%, and 57% respectively (Fig. 4A). In the case of U87.CD4.CCR5 cells, the percentage of subpopulations expressed on the surface were: 91%, 61%, 93%, 87%, 87%, and 52% respectively (Fig. 4B). The limited cell membrane labeling seen with ME mAb 45523 was reflected in our analysis by its percentage labeling (Fig. 4A, B). Conformations detected by mAbs in Fig. 4 were abundant on the cell surface, with around 20–40% of all conformations bound by each mAb on the cell surface alone. These conformations are also found internally, although the proportions vary as presented in Fig. 4. For example, CTC8, an NT-specific mAb, recognizes conformations that appear ~ 50% internally, with a surface colocalization coefficient of ~ 20% compared to a total colocalization coefficient of ~ 40%. However, the 45531-epitope targeting ECL1/ECL2 was detected primarily on the surface (80+%). Some mAbs may have overlapping conformations depending on which epitopes are available for binding on each CCR5 molecule. There was a significant proportion of internal conformations with recognizable CTC8 or 45523 epitopes (Fig. 4B) with p = 0.002 and < 0.001 respectively in U87.CD4.CCR5 cells. Differences between surface and total frequency in all other data sets were not statistically significant.

### Kinetics of CCR5 internalization and recycling

**Fig. 5:**
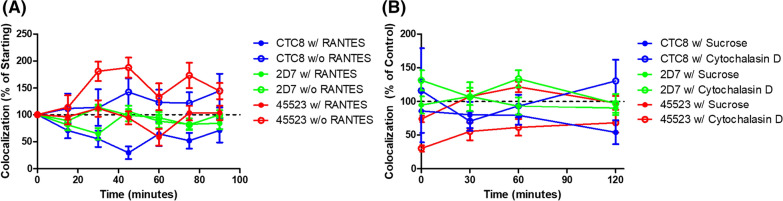
Endocytosis and recycling rates of CCR5 surface populations in U87.CD4.CCR5 cells: temperature versus RANTES trigger. U87.CD4 cells were transfected with CCR5-GFP. 24 h post-transfection, cells were divided into groups and treated with or without 100 nM RANTES at 37 °C (**A**). After 0, 15, 30, 45, 60, 75, or 90 min, cells were fixed and then stained with CTC8 (blue lines), 2D7 (green lines), or 45523 (red lines). Colocalization percentages first were normalized to the 0-min timepoint and graphed. To determine recovery rates, U87.CD4 cells were transfected with CCR5-GFP. 24 h post-transfection, cells were divided into groups and treated with RANTES for 90 min at 37 °C. RANTES was removed, and cells were treated with 1 µM cytochalasin D, 0.45 M sucrose, or left as a volume equivalent control for 0, 30, 60, or 120 min at 37 °C (**B**). After treatment, cells were fixed and stained with CTC8 (blue lines), 2D7 (green lines), or 45523 (red lines). Colocalization coefficients were calculated using ZEN Blue 2.3 software at ×1.3 zoom using 10 different cells and graphed as a percentage of the control (without treatment)

We next quantified rates of CCR5 internalization and recycling to better understand how different conformations are trafficked and redistributed (Fig. 5). CCR5 internalization was triggered by treating cells with native RANTES at 37 °C and comparing CCR5 surface conformation appearances over time to those without RANTES treatment. The dynamics of CCR5 population cycling (both on the surface and internally within the cells) should give insight into HIV-1’s entry potential depending upon the presence of selected CCR5 subpopulations. To measure RANTES-induced CCR5 endocytosis, we first quantified the localization of CCR5 conformations using mAbs against different CCR5 domains and their rates of internalization (Fig. 5A). For anti-NT, anti-ECL2, and anti-ME mAbs, RANTES caused strong internalization (solid symbols) compared with a 37 °C temperature trigger alone control experiment (open symbols). The mAbs specific for ME ECL1/2 (45523) and the NT (CTC8) showed a great increase in internalization in response to RANTES (red and blue symbols respectively). Interestingly, this was not the case with mAb 2D7, which recognizes a conformational epitope in ECL2, suggesting either a conformational change that occurs during internalization, or that this conformation more readily binds RANTES, leading to faster internalization and subsequent recycling. Cell surface CCR5 was reduced by ~ 50% after 30 min of a saturating concentration (100 nM) of RANTES (anti-NT and anti-ECL2 mAb, the blue and green symbols respectively), while anti-ME mAb (red symbols) required approximately 60 min to reach its maximum internalization (Fig. 5A). These differences need further investigation and may be related to different rates of CCR5 endocytosis, recycling, and binding affinity of RANTES to the cell surface [12, 53], but the data support the classical model for chemokine binding to CCR5 [7] that describes the chemokine core binding to extracellular regions of CCR5, while the N-terminal tail of RANTES interacts with the transmembrane domains. CCR5 was redistributed upon RANTES application. The analysis did not focus on competitive binding between RANTES and mAbs due to RANTES inducing internalization of CCR5, and only surface CCR5 events being measured. Next, we monitored recycling rates upon RANTES removal and CCR5 reappearance rates on the cell surface (Fig. 5B), using the same set of mAbs and staining procedure that were used in Fig. 5A. Figure 5B is a direct kinetics experiment continuation of the kinetics experiment presented in Fig. 5A.

Knowing that CCR5 internalization and recycling rates are regulated by actin polymerization and activation of small G proteins [54], we measured the present CCR5 epitopes available for mAb binding on the cell surface upon cytochalasin D (depolymerizes actin) addition (Fig. 5B), which prevents CCR5 internalization by blocking endocytosis [12, 54], and sucrose [12], which depletes cytoplasmic pools of clathrin by causing an accumulation of clathrin microcages on the inner cellular membrane as percentages of the volume-equivalent control (without treatment) as a continuation of the timepoints in Fig. 5A. Our rationale for this experimental effort was that actin filaments contribute to processes such as CCR5 endocytosis [51, 54], and we wanted to know which CCR5 populations were primarily affected by the blockage of these endocytic pathways. We selected antibodies targeting conformations that showed a high level of naturally internalized molecules in U87.CD4.CCR5 cells. Based on the results of Fig. 5, there did not seem to be any major differences between the untreated control sample (black dotted 100% line) and conformations labeled for the CTC8 or 2D7 epitopes, which indicates that recycling is not significantly dependent on these trafficking pathways (p > 0.05 at each time point). There also did not seem to be any major difference between the control and conformations labeled for the 45523-epitope treated with sucrose (p > 0.05 at each time point taken). However, there was a significant reduction in CCR5 surface recovery for conformations labeled with the ME 45523 antibody and treated with cytochalasin D until 120 min (p ≤ 0.001 at 0 min, 0.006 at 30 min, 0.02 at 60 min) (Fig. 5B).

### CCR5 conformations used for HIV-1 infection in primary cells

**Fig. 6:**
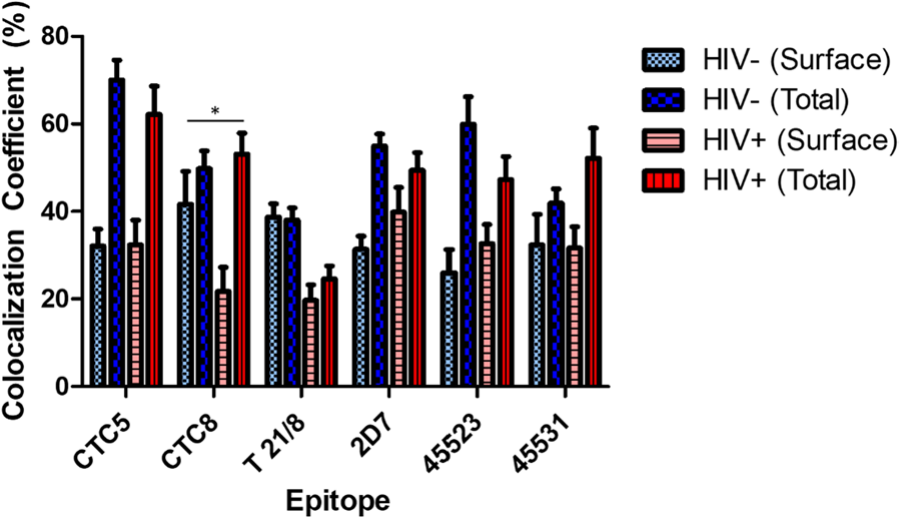
HIV-1 induction of CCR5 internalization. Evaluation of CCR5 subpopulations in non-infected versus HIV-1 infected primary cells. PBMCs isolated from whole blood were grown for 3 days in culture media containing IL-2 and PHA. Cells were cultured for 1 week (**A**) or infected for 3 h with HIV_BaL_ and cultured for 1 week post-infection (**B**) before being stained with one of six antibodies: CTC5 (NT), CTC8 (NT), T 21/8 (NT), 2D7 (ECL2), 45523 (ECL1/ECL2), or 45531 (ECL2). All primary antibodies were labeled with a secondary AlexaFluor 647-conjugated antibody. Cells were then permeabilized and labeled with an anti-CCR5 CT antibody, followed by a secondary DyLight 488-conjugated antibody. Cells were imaged and colocalization coefficients normalized to total DyLight (488 nm) and calculated using ZEN Blue 2.3 software at ×1.3 zoom using 10 different cells. This was repeated in a second donor for validation. Data was graphed using GraphPad Prism 9. *p < 0.05

To address which CCR5 epitopes (and by extension, which CCR5 conformations) are primarily used for HIV-1 infection, we identified a high CCR5 expressing PBMC donor and infected the cells with HIV-1_BaL_ for 1 week before staining and measuring CCR5 surface conformation frequency using the six mAbs previously used in the three cell line experiments. Colocalization coefficients were calculated with or without HIV-1 infection and compared to determine how HIV-1 affects CCR5 surface subpopulations (Fig. 6). Those conformations that had a greater reduction in surface density after HIV-1 challenge should be those more readily bound (and internalized) by the virus, resulting in a reduction in their surface density. For the most part, there was not a major change in the percentage of surface conformations after HIV-1 challenge, with only a notable drop (p = 0.033) in conformations with the CTC8 epitope. We confirmed this for a second donor (data not shown) that exhibited an even larger drop in surface CTC8 conformations (p < 0.001), while also exhibiting a drop in relative surface 45523 conformations (p = 0.001), although this could not be replicated in the first donor. Other differences between surface CCR5 conformation densities with or without HIV-1 infection were not significant. Total (permeabilized) colocalization remained relatively the same between infected and non-infected cells except for the T 21/8 epitope in the first donor, which showed lower total CCR5 compared to non-infected cells, although this could not be replicated in the second donor. The second donor showed no significant differences in total CCR5 with or without infection for any detectable conformations. PBMC culture supernatants were additionally checked for p24 via ELISA to ensure the cells were productively infected (data not shown). This was repeated with a second donor to validate the pattern (data not shown).

## Discussion

Our goal was to characterize the relation between the function and dynamics of CCR5 subpopulations with its implications for understanding their selective targeting by HIV-1 and for the development of more specific CCR5 inhibitors that will block CCR5 utilization by the virus. This study takes the first steps towards exploring the full potential of developing a new generation of CCR5 targeting drugs. The outcomes of this study were based on novel quantitative, 3D, temporal single molecule and super resolution imaging that distinguishes among molecular subpopulations of the CCR5 coreceptor.

Our work demonstrates that quantitative 3D Airyscan-based super-resolution visualization methodologies were indeed able to distinguish among CCR5 subpopulations qualitatively and quantitatively, as well as to characterize internalization rates of CCR5 subpopulations. Optical imaging and quantitative measurements of CCR5 subpopulations in this work showed the existence of distinct CCR5 conformational populations by contrasting surface and total labeling among different CCR5 mAbs. The existence of different and quantified CCR5 groups may contribute to the design of better CCR5 inhibitors and antiretroviral therapy in general. We analyzed the densities and spatial distributions of CCR5 populations in cell lines expressing higher or lower levels of CCR5 (JC10 vs JC53 cell lines) (Additional file 1: Fig. S1). As anticipated, competition with an HIV-1 envelope analog (FLSC IgG1) substantially reduced antibody binding, demonstrating the ability of our assay to differentiate between high- and low-expressing cell types. We next visualized differences between intracellular (after permeabilization) and surface localization of subpopulations within the high CCR5-expressing JC53 cell line. Visualization of total cellular events allowed us to determine the percentage of conformations existing internally by calculating the difference between total and surface labeling percentages for a given mAb (Fig. 3 and Additional file 2: Fig. S2). mAb 45523 captured a conformation whose surface expression in the U87.CD4 cell line was clearly diminished compared to other highly expressed CCR5 conformations. We visualized other CCR5 conformations in model cell lines that exhibit different localization and abundance patterns throughout the cell, supporting previous findings that subpopulations differ between cell types (Fig. 4) [12, 38]. Using the chemokine RANTES versus a natural temperature trigger for CCR5 internalization and changes among CCR5 populations on U87.CD4.CCR5 cells, it was also evident that responses to ligand engagement are conformation-specific (Fig. 5). RANTES promoted different engagement of CCR5 subpopulations and different responses between CCR5 conformations, in agreement with previous findings [42, 48, 52]. We additionally noted that only certain CCR5 subpopulations (those with the CTC8 epitope available for binding) were selectively internalized upon HIV-1 infection (Fig. 6). Altogether, our data show that CCR5 conformations are utilized by HIV-1 selectively. HIV-1 appeared to use CCR5 conformations most populated on the cell surface (eg., CTC8), confirming the paramount importance of the role of the CCR5 coreceptor in HIV-1 entry.

We then measured total versus surface CCR5 from individual pixel fluorescence intensities in donor primary cells relative to total CCR5. Visual and quantitative analyses demonstrated the existence of CCR5 conformations among six different CCR5 mAbs. The mAb against the 45531-epitope, targeting the ECL2, detected a conformation whose surface expression was higher (80%+) compared to other measured CCR5 conformations, which agrees with previous findings of others. Although with primary cells we detected CCR5 only via mAb staining, the above method allowed us to quantify changes in surface CCR5 subpopulations after virus treatment. However, there are other possibilities that can change mAb affinity, such as very prominent heterogeneity of CCR5 and cellular membrane processes, and membrane fixation, both of which we are currently investigating by live cell imaging.

The current findings underscore the ability of diverse CCR5 antibodies to identify CCR5 conformations with distinct functions and serve as a proof of principle for more thorough analyses in primary cells and humanized mouse tissues. For example, understanding the relevance of each CCR5 conformation to HIV-1 biology could be further advanced in vivo by using human CD34+ hematopoietic stem cell based humanized mice and targeting CD4+ T cells. This model has comparable numbers of CD4+ T cells to that of HIV-1 patients treated with cART [55].

This study addresses the question of how HIV-1_BaL_ utilizes different CCR5 subpopulations for entry. Future studies will focus on how other HIV-1 strains utilize CCR5 subpopulations. Future efforts will include an analysis of CCR5-virion interactions in T/F coreceptor usage and entry, which are not yet well understood. More specifically, they would identify possible differences between T/F and chronic viruses in the utilization of different CCR5 subpopulations to facilitate the rational design of effective HIV-1 transmission prevention strategies.

## Supplementary Information

**Additional file 1: Figure S1.** CCR5 expression and its suppression in two cells lines with different number of surfaces CCR5 molecules. JC10 and JC53 HeLa derivatives (**A**, **B**) were harvested from culture and transfected via electroporation with a plasmid expressing human CCR5-HA for greater visualization of CCR5 and the inhibition of mAb binding upon FLSC-IgG1 treatment. Cells were grown in culture media for 24 h, then stained with CTC8 (labeling the CCR5 NT) or 2D7 (labeling the CCR5 ECL2) antibodies before and after treatment with 5 µg/mL FLSC IgG1. CCR5 regions are labeled in red. Bar size—5 µm.

**Additional file 2: Figure S2.** Surface and total CCR5 staining patterns. JC53 HeLa derivatives were transfected with CCR5-GFP, grown in culture media for 24 h post-transfection and stained with 2D7 (ECL2) or 45531 (ECL2, Cholesterol-Rich Regions). Cells in panels labeled Total were permeabilized prior to staining to visualize surface plus intracellular CCR5 subpopulations. Primary antibodies were labeled with a secondary AlexaFluor 647-conjugated antibody. Cells were imaged using ZEN Blue 2.3 software at 1.3× zoom and the best representative cell was used for the image (**A**). White arrows indicate localization in cholesterol-rich areas in the total 45531, which appear predominantly on the cell surface. Quantified analysis of surface and total colocalization coefficients was done using the colocalization software in ZEN Blue 2.3, and graphs were produced using GraphPad Prism 9, in support of the visualization data (**B**). Bar size—5 µm.

## Data Availability

The datasets during and/or analyzed during the current study available from the corresponding author on reasonable request.
