# Supplementary figures and images for: Identifying CCR5 coreceptor populations permissive for HIV-1 entry and productive infection: implications for in vivo studies
Source: J Transl Med. 2022 Jan 24;20:39. doi: 10.1186/s12967-022-03243-8 (PMC8785515; doi:10.1186/s12967-022-03243-8)

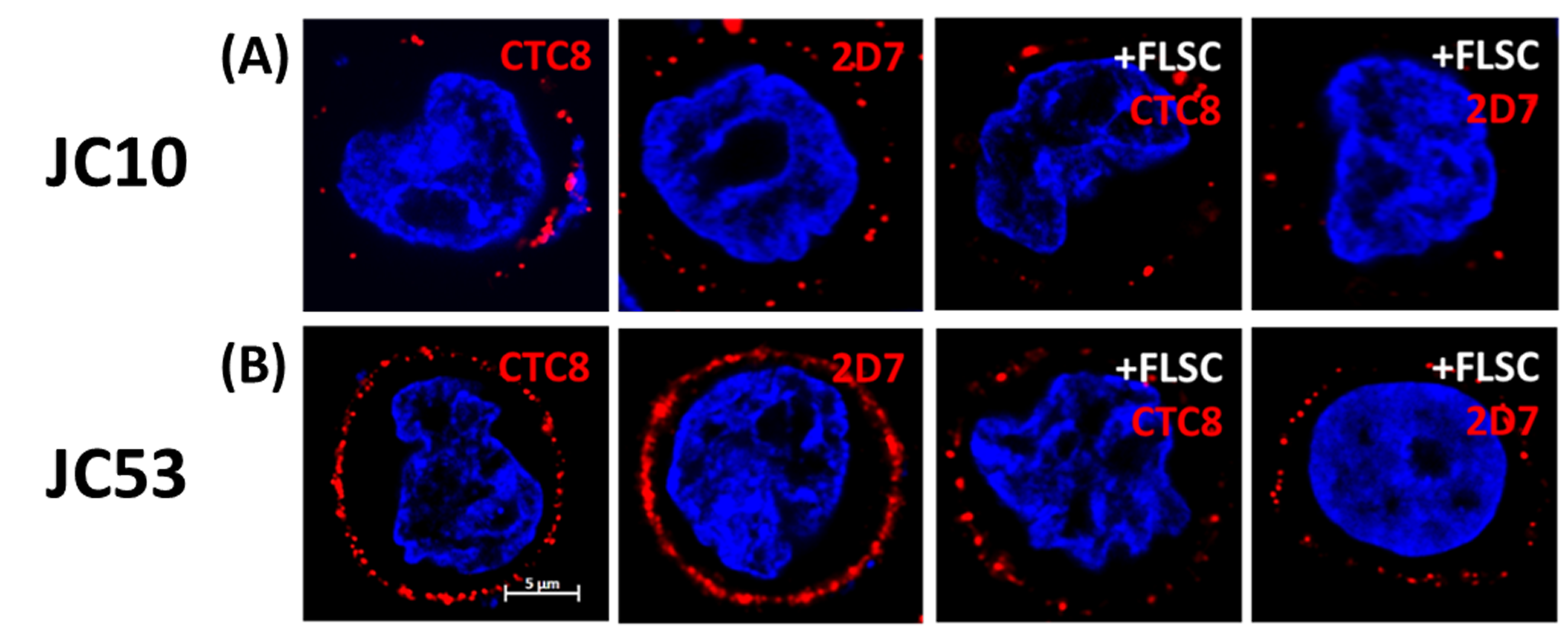

Supplement: Supplementary file 1 — Additional file 1: Figure S1. CCR5 expression and its suppression in two cells lines with different number of surfaces CCR5 molecules. JC10 and JC53 HeLa derivatives (A, B) were harvested from culture and transfected via electroporation with a plasmid expressing human CCR5-HA for greater visualization of CCR5 and the inhibition of mAb binding upon FLSC-IgG1 treatment. Cells were grown in culture media for 24 h, then stained with CTC8 (labeling the CCR5 NT) or 2D7 (labeling the CCR5 ECL2) antibodies before and after treatment with 5 µg/mL FLSC IgG1. CCR5 regions are labeled in red. Bar size—5 µm. [file 12967_2022_3243_MOESM1_ESM.tif]

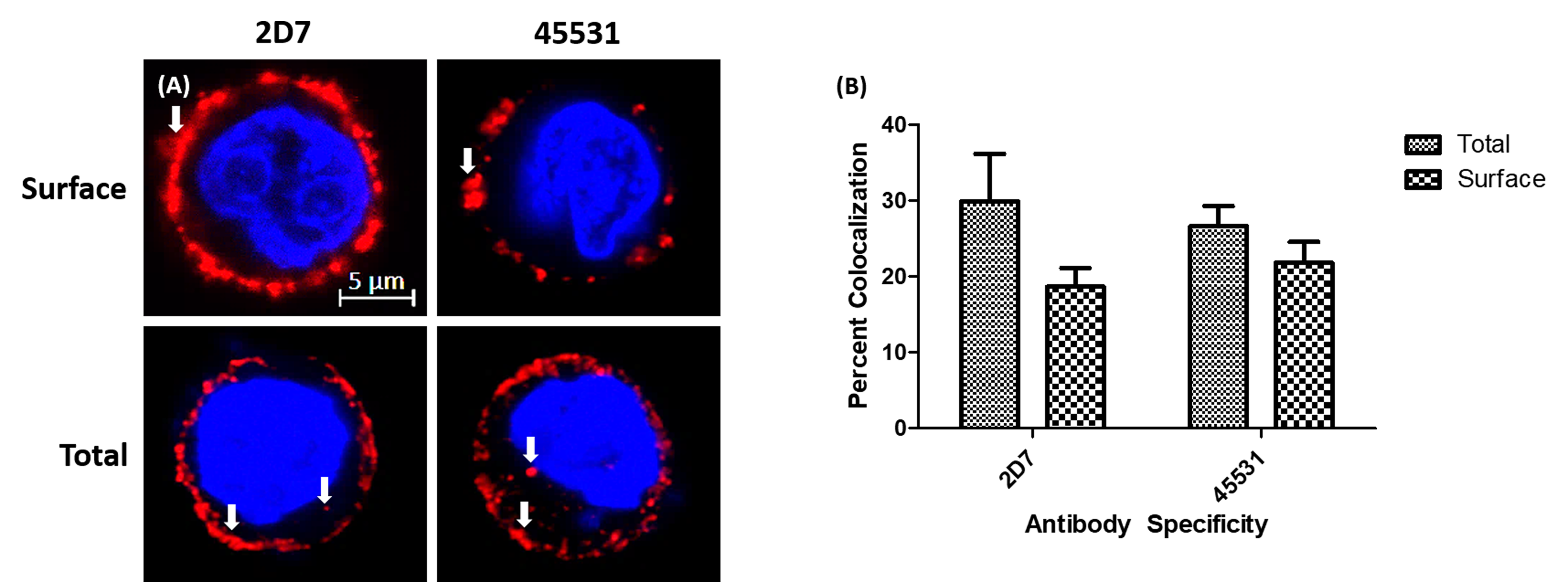

Supplement: Supplementary file 2 — Additional file 2: Figure S2. Surface and total CCR5 staining patterns. JC53 HeLa derivatives were transfected with CCR5-GFP, grown in culture media for 24 h post-transfection and stained with 2D7 (ECL2) or 45531 (ECL2, Cholesterol-Rich Regions). Cells in panels labeled Total were permeabilized prior to staining to visualize surface plus intracellular CCR5 subpopulations. Primary antibodies were labeled with a secondary AlexaFluor 647-conjugated antibody. Cells were imaged using ZEN Blue 2.3 software at 1.3× zoom and the best representative cell was used for the image (A). White arrows indicate localization in cholesterol-rich areas in the total 45531, which appear predominantly on the cell surface. Quantified analysis of surface and total colocalization coefficients was done using the colocalization software in ZEN Blue 2.3, and graphs were produced using GraphPad Prism 9, in support of the visualization data (B). Bar size—5 µm. [file 12967_2022_3243_MOESM2_ESM.tif]
